# Evaluation of Residual Monomers Eluted from Pediatric Dental Restorative Materials

**DOI:** 10.1155/2021/6316171

**Published:** 2021-09-16

**Authors:** Tugba Bezgin, Ceren Cimen, Nurhan Ozalp

**Affiliations:** Department of Pediatric Dentistry, Faculty of Dentistry, Ankara University, Besevler, 06500 Ankara, Turkey

## Abstract

Unreacted monomers eluted from resin-based restorative materials have been considered a reason of local and systemic adverse reactions. This study was designed to determine the effect of finishing and polishing procedures on the elution of Bis-GMA, TEGDMA, UDMA, and HEMA monomers from compomer and bulk-fill composite resins. Bulk-fill composite (3M ESPE GmbH, Seefeld, Germany) and compomer (Dentsply DeTrey GmbH, Konstanz, Germany) specimens with 3 × 4 mm diameters were prepared. The specimens were randomly divided into two groups, and finishing-polishing procedures were applied only to the experimental groups. Release of residual monomers was analyzed by using High-Performance Liquid Chromatography (HPLC) after 24, 48, and 72 hours. Repeated measures ANOVA and Tukey post hoc tests were used for comparisons. Finishing and polishing procedures had a significant effect on reducing the quantity of UDMA release in the Filtek™ Bulk Fill composite and Bis-GMA, HEMA, and TEGDMA in the Dyract XP compomer (*p* < 0.05). The restorative materials investigated here are not chemically stable after polymerization, and concentrations of eluted monomers may reach critical toxicity levels even after one restoration placement. Finishing and polishing procedures are mandatory to reduce residual monomers.

## 1. Introduction

In the early 1990s, polyacid-modified composite resins (compomers) are introduced to combine the superior mechanical properties of composite resins and fluoride release of glass-ionomer cements to overcome the disadvantages such as water sensitivity and physical strength of glass ionomer cements [1]. In recent years, bulk-fill composite resins are developed with the promise of placing single bulk increment up to 4-5 mm, thus providing lesser risk of contamination and reduced chair time which are important while working on pediatric patients [2].

Resin-based restorative materials consist of an organic polymerizable matrix, filler materials, molecules initiating the polymerization reaction, and silane coupling agents [3]. Mainly used constituents of the organic matrix are cross-linking dimethacrylates such as bisphenol-A-glycidyl dimethacrylate (Bis-GMA), triethylene glycol dimethacrylate (TEGDMA), urethane dimethacrylate (UDMA), and hydroxyethyl methacrylate (HEMA) [4].

The polymerization of resin-based restorative materials is the end product of a chemical reaction between the methacrylate resin monomers that results in the formation of a rigid and highly cross-linked polymer network [5]. It is known that atmospheric oxygen inhibits the polymerization of monomers. This results in an “oxygen inhibition layer (OIL)” on the surface of resin-based restorative materials which are rich in unreacted monomers [6]. The degree of conversion of monomer to polymer in resin-based restorative materials varies between 55% and 80%, and this rate decreases to 35% in the presence of an OIL [7]. It has been reported that the degree of conversion further increased to nearly 95% when the OIL was removed by finishing and polishing techniques [8]. OIL could be minimized by blocking air contact with the use of matrix strips or glycerin before curing; however, since there is oxygen already present within the resin material, the most effective method to eliminate the OIL is to finish and polish the surface after curing [8, 9]. Finishing and polishing procedures are also essential for the elimination of the resin-rich outer surface [8–12].

Unreacted monomers eluted from resin-based restorative materials have been considered a reason of local and systemic adverse reactions, such as estrogenicity, cytotoxic effects on cell metabolism, genotoxicity, and mutagenicity [13, 14]. Quantity of the residual monomers in resin-based restorative materials was investigated in many studies [15–17]. However, there is no study in the literature investigating the effect of finishing and polishing procedures on monomer elution from resin-based restorative materials. Therefore, the present study was aimed at evaluating the effect of finishing and polishing procedures on monomer elution in the compomer that is commonly used in pediatric patients and in bulk-fill restorative resins which has become popular in pediatric dentistry, using High-Performance Liquid Chromatography (HPLC). The null hypothesis to be tested was that finishing and polishing procedures do not cause difference at elution of residual unreacted monomers from resin-based restorative materials.

## 2. Materials and Methods

### 2.1. Specimen Preparation

This study has followed the CRIS guidelines for in vitro studies as discussed in the 2014 concept note. Two types of resin-based restorative materials, bulk-fill composite (3M ESPE GmbH, Seefeld, Germany) and compomer (Dentsply DeTrey GmbH, Konstanz, Germany), in A2 shade were used. Power analysis indicated that a minimum of 60 teeth were required based on an effect size of 0.5, an alpha significance level of 5% (0.05), and a beta of 20% (0.20) to achieve an 80% power to detect a difference of 20% based on a previously conducted research [16]. The compositions of the tested restorative materials are given in Table 1.

The test specimens were prepared by using a cylindrical plexiglass mold of 3 mm in diameter and 4 mm in height [16]. The volume of test specimens was calculated as 28.3 mm^3^, which approximately simulates the mesio-occluso-distal cavity volume of primary teeth [18]. Bulk-fill composite resins were applied as a single bulk increment of 4 mm, and compomers were applied in two increments with the height of 2 mm. A plexiglass mold is covered with Mylar strips (SNA, Universal Strips, Cologne, Germany) and with 1 mm thick glass slides at the top and bottom. Glass slides were then finger-pressed to the height of the mold to extrude the excess material. Bulk-fill composite specimens were cured by using a LED light curing unit (Elipar S10; 3M Unitek, Monrovia, Calif) with irradiance of 1000 mW/cm^2^ for 20 seconds. Every increment of compomer specimens was cured for 10 seconds according to the manufacturer's instructions. The consistency of the curing light intensity was verified using a radiometer (PMA2100, Solar Light, Pennsylvania, USA) for each irradiation. After curing, the specimens were randomly divided into two groups, and finishing-polishing procedures were applied to the experimental groups only (Filtek™ Bulk Fill+ (*n* = 15), Dyract XP+ (*n* = 15)). No finishing or polishing procedures were applied to the control groups (Filtek™ Bulk Fill- (*n* = 15), Dyract XP- (*n* = 15)).

In the experimental groups, finishing procedures were performed with a 12-fluted carbide finishing bur (Hager & Meisinger GmbH, Neuss, Germany) in a high-speed handpiece under water cooling. The tungsten carbide burs were changed every 4 specimens. The specimens were then polished with coarse, medium, fine, and superfine grit Sof-Lex discs (3M ESPE, St. Paul, MN, USA) with a low-speed handpiece for 15 seconds, respectively. Each disc was discarded after each use. After each step of polishing, all specimens were rinsed with water for 10 seconds and air dried for 5 seconds. All specimen preparations and finishing and polishing procedures were carried out by the same investigator to provide standardization.

Each specimen was immediately immersed in amber-colored HPLC vials containing 1.5 mL 75% ethanol/water solution and stored at room temperature [16]. Fifteen test specimens were divided into 3 groups, and each of the 5-specimen group was retained in the solution for 24, 48, and 72 hours, respectively. Samples of 0.5 mL ethanol/water were collected from each vial at the end of the observation periods.

### 2.2. HPLC Analysis

The analysis was performed by using the HPLC system (Agilent Technologies, Palo Alto, CA) with a C18 column (150 × 4.6 mm; 5 *μ*m, ACE, Aberdeen, Scotland). Standard solutions of HEMA, Bis-GMA, UDMA, and TEGDMA (Sigma-Aldrich, St. Louis, MO, USA) were used for calibration. 5, 10, 25, 50, and 100 *μ*g/ml solutions of each monomer were prepared and injected into the HPLC system. The injection volume was 10 *μ*l on the column. The mobile phase was a solution of 80% acetonitrile (Sigma-Aldrich, St. Louis, MO, USA) and 20% water. UV detection was performed at 204 nm for HEMA and 193 nm for HEMA, TEGDMA, and UDMA. A calibration curve for each monomer was constructed from the injection standard solutions as an external standard. Correlation coefficients and linear range mathematical equations of monomers were obtained by linear regression analysis of concentration in standard solutions. Retention times, correlation coefficients, regression equations of the calibration curves, limit of detection (LOD), and limit of quantitation (LOQ) values of the monomers are given in Table 2.

### 2.3. Statistical Analysis

The statistical analyses were performed with R Studio (RStudio: Integrated Development for R. RStudio, Inc., Boston, MA). The results of the Shapiro-Wilk normality test showed that the data was normally distributed. Therefore, repeated measures ANOVA and Tukey post hoc tests were used for comparisons. The confidence interval was set to 95%, and values of *p* < 0.05 were considered to be statistically significant.

## 3. Results

**Figure 1:**
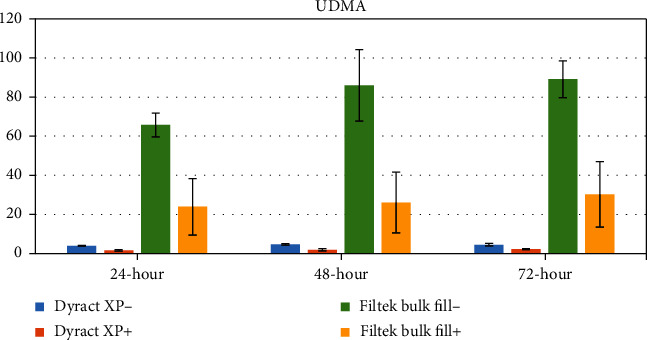
Graphical view of UDMA release from tested restorative materials.

**Figure 2:**
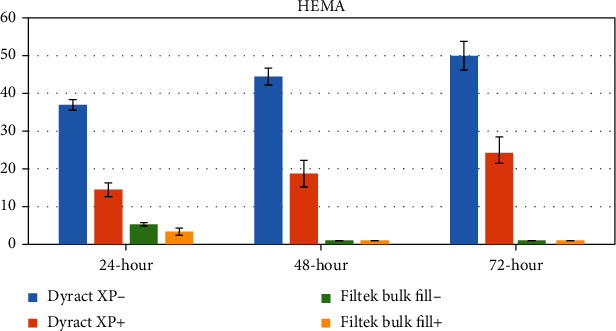
Graphical view of HEMA release from tested restorative materials.

**Figure 3:**
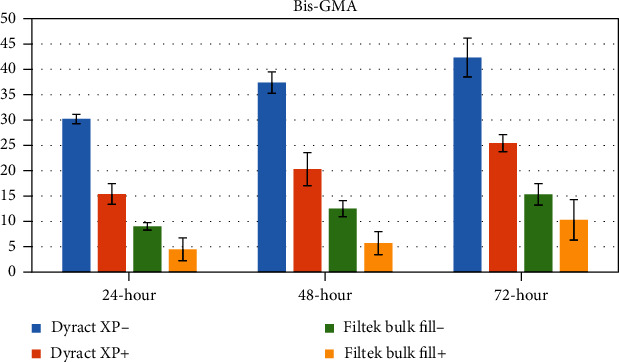
Graphical view of Bis-GMA release from tested restorative materials.

**Figure 4:**
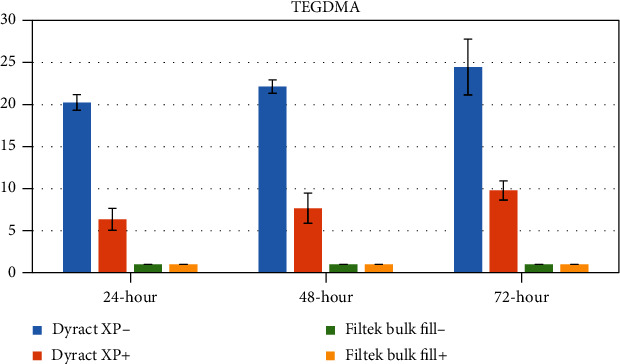
Graphical view of TEGDMA release from tested restorative materials.

The concentrations of eluted monomers were determined as a mean ± standard deviation (*μ*g/mL) using peak areas. The mean values and standard deviations of the monomers eluted from test specimens are shown in Tables 3–6 and Figures 1–4.

### 3.1. UDMA

Elution of UDMA was reduced after finishing and polishing procedures at all time periods in both Filtek™ Bulk Fill groups and Dyract XP groups; meanwhile, the decrease was significant only in Filtek™ Bulk Fill groups (*p* < 0.001). The cumulative quantities of UDMA in Filtek™ Bulk Fill- at the 48^th^ and 72^nd^ hours were significantly higher than those at the 24^th^ hour (*p* < 0.001) (Table 3, Figure 1).

### 3.2. HEMA

HEMA elution dropped significantly in Dyract XP+ when compared to Dyract XP- at all time periods (*p* < 0.001). The highest cumulative quantity of HEMA was detected at the 72^nd^ hour in Dyract XP-, which is significantly higher than the quantity at the 24^th^ hour (*p* < 0.05). Elution of HEMA was not detected in Filtek™ Bulk Fill- and Filtek™ Bulk Fill+ at the 48^th^ and 72^nd^ hours. The decrease in the elution of HEMA in Filtek Bulk Fill- was not significant in comparison to that in Filtek Bulk Fill+ (Table 4, Figure 2).

### 3.3. Bis-GMA

The reduction in the quantity of Bis-GMA was not significant for both Filtek™ Bulk Fill- and Filtek™ Bulk Fill+ at any time period. However, Bis-GMA elution was significantly lower in Dyract XP+ in comparison to Dyract XP- at all time periods (*p* < 0.05; *p* < 0.001). The cumulative quantity of Bis-GMA in Dyract XP- at the 72^nd^ hour significantly increased when compared to that at the first 24-hour span (*p* < 0.05) (Table 5, Figure 3).

### 3.4. TEGDMA

There was no release of TEGDMA in both Filtek™ Bulk Fill composite groups at any time periods. In Dyract XP groups, finishing and polishing procedures led to a significant decrease in the quantity of TEGDMA at all time periods (*p* < 0.05) (Table 6, Figure 4).

## 4. Discussion

In the present study, HPLC analysis demonstrated that residual monomers were leached from resin-based restorative materials and the quantity of residual monomers could be reduced with finishing and polishing procedures.

Resin-based restorative materials may cause hazards due to the release of unreacted monomers after polymerization. It was reported that the resin monomers are able to increase the amount of reactive oxygen species and oxidative stress which results in apoptosis of the cell. They also have been found to be related to DNA strand breaks, caspase activation, and delay in the cell cycle [14]. In many studies, toxic doses of monomers have been investigated and various results have been obtained by the test method and cell type dependence. The toxicity for the following monomers was ranked as Bis‐GMA > UDMA > TEGDMA > HEMA (least toxic) [19, 20]. In another study, exposure of dental pulp cells to Bis-GMA at concentrations of 0.075 mmol/L markedly affected the viable cell number with 40% of inhibition [19]. Reichl et al. [21] reported the concentration that causes 50% reduction of cell viability which is named half maximal effect concentration (EC_50_) of UDMA and Bis-GMA as 0.106 mmol/L and 0.087 mmol/L on human gingival fibroblasts, respectively. Toxic concentration_50_ (TC_50_) of HEMA ranged from 3.6 mmol/L to 11.2 mmol/L with different cell lines in various studies [22–24]. The effective dose that reduced the number of cell viability to 50% for TEGDMA was reported as 0.26 mmol/L on human pulp fibroblasts [19] and 3.46 mmol/L on human gingival fibroblasts [21]. In our study, Bis-GMA concentration in Dyract XP- at the 72^nd^ hour was found either equal or greater than the toxic concentrations obtained in some previous studies [21, 22, 25]. UDMA concentrations in Filtek™ Bulk Fill- for all retention times were also higher than the toxic concentration reported in the study of Reichl et al. [21]. However, after finishing and polishing procedures were applied, neither of the monomer concentrations in groups was above the toxic doses.

HEMA and Bis-GMA were detected in both of the restorative materials tested. However, these monomers were not listed as ingredients in the Material Safety Data Sheet (MSDS) of the products. Manufacturers are not obligated to reveal the components with concentrations lower than 1% in their products as trade secret. Furthermore, it was shown that ingredients in MSDS are sometimes insufficient [26, 27]. Botsali et al. [28] also confirmed the presence of HEMA in the Dyract XP compomer. Another reason for HEMA elution in this study could be that it was a degradation product from UDMA, which is an ingredient in both restorative materials [29]. Therefore; HEMA elution detected from Filtek Bulk Fill- and Filtek Bulk Fill+ groups at the 24^th^ hour may have been going down below the detection limits at the 48^th^ and 72^nd^ hours. However, to the best of our knowledge, no previous study has ever investigated monomer elution from the Filtek™ Bulk Fill composite with or without finishing and polishing procedures by using HPLC analysis, which makes it difficult for us to compare the results of our study.

Time is also a significant factor on monomer elution. Some studies have reported that acute release of monomers occurs in the first 24 hours [30, 31]. However, some recent researches have shown that monomer elution is not completed within the first 24 hours and leaching in certain monomers continued for a longer time [16, 31]. Therefore, 72 hours of elution was investigated in this study and found that monomers mostly leached in the first 24 hours which is consistent with previous studies, but the elution of UDMA in Filtek™ Bulk Fill- significantly increased in time. Similarly, the elution of HEMA and Bis-GMA from Filtek™ Bulk Fill- was also higher at the 72^nd^ hour when compared to the level of elution from Dyract XP- at the 24^th^ hour. However, this increase in the quantity of monomers over time was not observed in restorative material groups when finishing and polishing procedures were applied. Therefore, since unreacted monomers were removed by finishing and polishing agents, monomer elution in Filtek™ Bulk Fill+ and Dyract XP+ was observed in lower quantities and it did not increase significantly over time. Also, it is possible to say that the surface of test specimens was still rich in unreacted monomers when finishing and polishing were not performed, although clinicians usually think that the use of matrix strips prevented the formation of an oxygen inhibition zone. In line with previous studies [11, 12], this study showed that Mylar strips may minimize the formation OIL, but finishing and polishing procedures are still essential for the elimination of the resin-rich outer layer that can be the source of the unreacted monomers eluted to the oral cavity.

Finishing and polishing procedures had a significant effect on reducing the quantity of UDMA in the Filtek™ Bulk Fill composite and Bis-GMA, HEMA, and TEGDMA in the Dyract XP compomer. Because of these differences, the null hypothesis had to be rejected. The test specimens in this study simulated the mesio-occluso-distal cavity volume of primary teeth [18]. Therefore, the quantity of elution demonstrated in this study was from a single restoration. The quantity of unreacted monomers may reach dangerous levels when more than one restoration is performed in the same treatment session. Furthermore, compomer and bulk-fill composites are frequently used in dental restorations of pediatric patients, and the restorations are expected to last for a reasonable time. There are few studies that investigate the long-term elution of monomers for 1, 3, and 12 months [16, 17]. However, long-term effects of residual monomers on biocompatibility are still unclear. Due to constant flow of saliva in oral environment, it is believed that monomer concentrations may not reach cumulative values found in this study, whereas long-term chronic exposure and systemic adverse effects must also be considered when assessing the potential toxicity of the eluted compounds. Thus, finishing and polishing procedures play an important role in the elimination of unreacted monomers to the highest possible extent to prevent health effects from long-term exposure.

This research has several limitations. Firstly, only one brand of the compomer and bulk-fill composite was used for the assessment of monomer elution. The differences in composition of resin-based restorative materials could result in variability at release of amount and type of monomers. Secondly, finishing and polishing procedures were implemented using 12-fluted carbide finishing bur and Sof-Lex discs. It was reported that the use of carbide burs and Sof-Lex discs provided the smoothest surface [32]; therefore, they are preferred in this study. However, further in vitro researches may focus the amount of monomer elution by using different restorative materials and finishing-polishing techniques.

According to the results of the present study, the elution of residual monomers was higher if finishing and polishing were not performed. The bulk-fill composite showed lesser monomer elution compared to the compomer restoration except UDMA elution. In addition, the bulk-fill composites have the advantage of shorter chair time which can be an important factor in pediatric dentistry. Therefore, it can be concluded that this type of restorative material can be a good alternative for pediatric patients [33]. However, long-term clinical studies are needed to evaluate its success.

## 5. Conclusion

1. Since the results of the present study demonstrated that the restorative materials investigated here are not chemically stable after polymerization and concentrations of eluted monomers may reach critical toxicity levels even after one restoration placement, further research is needed to understand potential long-term toxicity of resin-based restorative materials, especially for pediatric patients
2. This study showed that Mylar strips did not prevent the formation of the oxygen inhibition layer, and finishing-polishing is still essential for the elimination of the resin-rich outer layer that can be the source of the unreacted monomers eluted to the oral cavity
3. When placing multiple restorations at a single session, it is highly recommended to follow the instructions of manufacturers during polymerization and apply finishing and polishing procedures

## Figures and Tables

**Table 1:** Restorative materials used in the study.

Material type	Material	Composition	Manufacturer
Compomer	Dyract XP compomer	(i) Urethane dimethacrylate (UDMA) (ii) Carboxylic acid-modified dimethacrylate (TCB resin) (iii) Triethylene glycol dimethacrylate (TEGDMA) (iv) Trimethacrylate resin (TMPTMA) (v) Dimethacrylate resins (vi) Camphorquinone (vii) Ethyl-4(dimethylamino)benzoate (viii) Butylated hydroxytoluene (BHT) (ix) UV stabilizer (x) Strontium-alumino-sodium-fluoro-phosphor-silicate glass (xi) Highly dispersed silicon dioxide (xii) Strontium fluoride (xiii) Iron oxide pigments and titanium oxide pigments	Dentsply DeTrey GmbH, Konstanz, Germany
Bulk-fill composite	Filtek™ Bulk Fill composite	(i) Aromatic dimethacrylate (AUDMA) (ii) Addition-fragmentation monomers (AFM) (iii) UDMA (iv) 1,12-Dodecanediol dimethacrylate (DDDMA) (v) Nonagglomerated/nonaggregated 20 nm silica filler (vi) Nonagglomerated/nonaggregated 4 to 11 nm zirconia filler (vii) Aggregated zirconia/silica cluster filler (viii) Ytterbium trifluoride filler consisting of agglomerate 100 nm particles	3M ESPE GmbH, Seefeld, Germany

**Table 2:** Regression equations of the calibration curves, detection limits, and retention times of the monomers.

	Regression equation	*R* ^2^	LOD (*μ*g/mL)	LOQ (*μ*g/mL)	Retention time (minute)
Bis-GMA	*y* = 0.0148*x* − 1.7497	0.9978	0.680	2.053	2.85
TEGDMA	*y* = 0.0229*x* − 1.012	0.9997	0.788	2.363	2.32
HEMA	*y* = 0.0235*x*–19.753	0.9999	0.482	1.445	1.82
UDMA	*y* = 0.0351*x*–0.1227	0.9999	0.412	1.236	2.59

LOD: limit of detection; LOQ: limit of quantitation.

**Table 3:** Mean values and standard deviations (±SD) of UDMA (*μ*g/mL) eluted from restorative materials.

	Dyract XP-	Dyract XP+	Filtek™ Bulk Fill-	Filtek™ Bulk Fill+
24hours	3.981 ± 0.260^aA^ (0.0084)	1.630 ± 0.348^aA^ (0.0034)	65.682 ± 6.078^bA^ (0.1395)	23.898 ± 14.356^cA^ (0.0507)
48 hours	4.746 ± 0.354^aA^ (0.01)	1.993 ± 0.533^aA^ (0.0042)	85.974 ± 18.299^bB^ (0.1826)	26.090 ± 15.577^cA^ (0.0554)
72 hours	4.547 ± 0.592^aA^ (0.0096)	2.304 ± 0.329^aA^ (0.0048)	89.101 ± 9.325^bB^ (0.1893)	30.212 ± 16.750^cA^ (0.0641)

Different superscript uppercase letters indicate a significant difference (*p* < 0.05) within the same column; different superscript lowercase letters indicate a significant difference (*p* < 0.05) within the same row (Tukey post hoc test). The numbers in parentheses are expressed as mmol/L.

**Table 4:** Mean values and standard deviations (±SD) of HEMA (*μ*g/mL) eluted from restorative materials.

	Dyract XP-	Dyract XP+	Filtek™ Bulk Fill-	Filtek™ Bulk Fill+
24 hours	36.939 ± 1.413^bA^ (0.2838)	14.473 ± 1.805^aA^ (0.1112)	5.281 ± 0.481^a^ (0.0405)	3.344 ± 0.943^a^ (0.0256)
48 hours	44.418 ± 2.240^aAC^ (0.3413)	18.769 ± 3.518^bA^ (0.1442)	Below LOD	Below LOD
72 hours	49.939 ± 3.811^aBC^ (0.3837)	24.238 ± 2.504^bA^ (0.1862)	Below LOD	Below LOD

Different superscript uppercase letters indicate a significant difference (*p* < 0.05) within the same column; different superscript lowercase letters indicate a significant difference (*p* < 0.05) within the same row (Tukey post hoc test). The numbers in parentheses are expressed as mmol/L. LOD: limit of detection.

**Table 5:** Mean values and standard deviations (±SD) of Bis-GMA (*μ*g/mL) eluted from restorative materials.

	Dyract XP-	Dyract XP+	Filtek™ Bulk Fill-	Filtek™ Bulk Fill+
24 hours	30.209 ± 0.895^bA^ (0.0589)	15.386 ± 2.058^aA^ (0.03)	8.996 ± 0.721^aA^ (0.017)	4.482 ± 2.255^aA^ (0.0087)
48 hours	37.401 ± 2.135^bAC^ (0.0729)	20.306 ± 3.261^cdA^ (0.0396)	12.509 ± 1.580^adA^ (0.0244)	5.674 ± 2.289^aA^ (0.011)
72 hours	42.312 ± 3.823^bBC^ (0.0825)	25.435 ± 1.699^cdA^ (0.0498)	15.317 ± 2.113^adA^ (0.0298)	10.278 ± 4.011^aA^ (0.02)

Different superscript uppercase letters indicate a significant difference (*p* < 0.05) within the same column; different superscript lowercase letters indicate a significant difference (*p* < 0.05) within the same row (Tukey post hoc test). The numbers in parentheses are expressed as mmol/L.

**Table 6:** Mean values and standard deviations (±SD) of TEGDMA (*μ*g/mL) eluted from restorative materials.

	Dyract XP-	Dyract XP+	Filtek™ Bulk Fill-	Filtek™ Bulk Fill+
24 hours	20.249 ± 0.943^aA^ (0.0707)	6.342 ± 1.310^bA^ (0.022)	Below LOD	Below LOD
48 hours	22.137 ± 0.806^aA^ (0.0773)	7.667 ± 1.794^bA^ (0.0267)	Below LOD	Below LOD
72 hours	24.476 ± 3.323^aA^ (0.0854)	9.791 ± 1.145^bA^ (0.0341)	Below LOD	Below LOD

Different superscript uppercase letters indicate a significant difference (*p* < 0.05) within the same column; different superscript lowercase letters indicate a significant difference (*p* < 0.05) within the same row (Tukey post hoc test). The numbers in parentheses are expressed as mmol/L. LOD: limit of detection.

## Data Availability

All data of the present article are available on request by contacting the corresponding author.
